# Carbapenem Resistance Determinants Acquired through Novel Chromosomal Integrations in Extensively Drug-Resistant Pseudomonas aeruginosa

**DOI:** 10.1128/AAC.00289-21

**Published:** 2021-06-17

**Authors:** Jessin Janice, Nicholas Agyepong, Alex Owusu-Ofori, Usha Govinden, Sabiha Yusuf Essack, Ørjan Samuelsen, Arnfinn Sundsfjord, Torunn Pedersen

**Affiliations:** a Norwegian National Advisory Unit on Detection of Antimicrobial Resistance, Department of Microbiology and Infection Control, University Hospital of North Norway, Tromsø, Norway; b Department of Medical Biology, Faculty of Health Sciences, UiT The Arctic University of Norway, Tromsø, Norway; c Antimicrobial Research Unit, School of Health Sciences, University of KwaZulu-Natal, Durban, South Africa; d Department of Clinical Microbiology, School of Medicine and Dentistry, Kwame University of Science and Technology, Kumasi, Ghana; e Department of Pharmacy, Faculty of Health Sciences, UiT The Arctic University of Norway, Tromsø, Norway

**Keywords:** XDR *Pseudomonas aeruginosa*, Tn*402*-type integron, genomic island, region of genome plasticity, DIM carbapenemase, IMP carbapenemase

## Abstract

Two novel *bla*_DIM-1_- or *bla*_IMP-1_-containing genomic islands (GIs) were discovered by whole-genome sequence analyses in four extensively drug-resistant (XDR) Pseudomonas aeruginosa isolates from inpatients at a tertiary hospital in Ghana. The strains were of sequence type 234 (ST234) and formed a phylogenetic clade together with ST111, which is recognized as a global high-risk clone. Their carbapenem resistance was encoded by two Tn*402*-type integrons, In1592 (*bla*_DIM-1_) and In1595 (*bla*_IMP-1_), both carrying complete *tni* mobilization modules. In1595 was bound by conserved 25-bp inverted repeats (IRs) flanked by 5-bp direct repeats (DRs) associated with target site duplication. The integrons were embedded in two GIs that contained cognate integrases and were distinguished by a lower GC content than the chromosomal average. PAGI-97A (52.659 bp; In1592), which encoded a P4-type site-specific integrase of the tyrosine recombinase family in its 3′ border, was integrated into tRNA-Pro(ggg) and bracketed by a 49-bp perfect DR created by 3′-end target duplication. GIs with the same structural features, but diverse genetic content, were identified in 41/226 completed P. aeruginosa genomes. PAGI-97B (22,636 bp; In1595), which encoded an XerC/D superfamily integrase in its 5′ border, was inserted into the small RNA (sRNA) PrrF1/PrrF2 locus. Specific insertions into this highly conserved locus involved in iron-dependent regulation, all leaving PrrF1 intact, were identified in an additional six phylogenetically unrelated P. aeruginosa genomes. Our molecular analyses unveiled a hospital-associated clonal dissemination of carbapenem-resistant ST234 P. aeruginosa in which the XDR phenotype resulted from novel insertions of two GIs into specific chromosomal sites.

## INTRODUCTION

Pseudomonas aeruginosa is a significant cause of nosocomial infections, particularly in patients with compromised immunity. The bacterium is diverse and adaptable, widespread in nature, and readily acquires resistance to antibiotics (1, 2). Due to inherent as well as acquired resistance against several classes of antibiotics, carbapenems are increasingly used in the treatment of P. aeruginosa infections. The emergence of extensive drug resistance in P. aeruginosa has led to loss in the efficacy of almost all currently available antibiotics, including that of carbapenems (3).

Carbapenem resistance in P. aeruginosa may be attributed to one or a combination of three mechanisms, i.e., reduced permeability due to porin alteration or loss, increased efflux, and the expression of β-lactamases (4). Metallo-β-lactamases (MBLs) confer resistance to most β-lactams (including carbapenems), except for aztreonam (5). In P. aeruginosa, commonly identified MBLs include the VIM, IMP, and NDM families (6–8). The spread of carbapenem resistance is conveyed by horizontal gene transfer (HGT), which includes transposition and conjugation, and various mobile genetic elements carrying MBL genes have been identified (6, 9, 10). A majority of MBLs are contained as gene cassettes embedded in integron structures (9), which increase the genetic flexibility by mobilization of single resistance genes by site-specific recombination (11). Integrons themselves can be mobilized and transferred between cells by genetic hitchhiking through plasmids, transposons, or integrative and conjugative elements (ICEs) (12). Class 1 integrons, which are extensively found in clinical isolates, are associated with functional or nonfunctional transposons derived from the Tn*402*/Tn*5090* family (13).

P. aeruginosa has a nonclonal population structure dominated by a limited number of successful epidemic clones (14, 15). These are distributed worldwide and have been denominated high-risk clones. They have multidrug-resistant (MDR) and extensively drug-resistant (XDR) phenotypes associated with over 60 different β-lactamase variants, including MBLs (16). The most relevant ones include sequence type 235 (ST235), ST111, ST233, ST244, ST357, ST308, ST175, ST277, ST654, and ST298 (17, 18). These STs are phylogenetically diverse, indicating independent evolution with recombination playing an important role (14). P. aeruginosa core genome phylogeny has provided evidence for five main groups, with frequent intragroup and limited intergroup recombination (19–21). Main groups 1 and 2, represented by the reference genomes PAO1 and PA14, respectively, are predominantly associated with clinical isolates (21, 22).

The genome of P. aeruginosa is mosaic, consisting of highly conserved core components with 0.5 to 0.7% sequence diversity, interrupted by strain-specific blocks acquired by HGT (23–25). These regions of genome plasticity (RGP) are located in a limited number of chromosomal sites and render the pangenome large and complex (26–28). Genetic elements embedded in the RGP, often referred to as genomic islands (GIs), contain strain-specific compositions of accessory genes, including a high fraction without P. aeruginosa homologs (29). GIs (∼10 to 500 kb) are characteristically located at specific sites in the bacterial chromosome, often near a tRNA gene, are flanked by direct repeats (DRs) resulting from the integration event, and harbor phage- and/or plasmid-associated genes, including an integrase that is responsible for their integration/excision (30).

Mobilizable GIs, including ICEs, have been suggested as reservoirs of transmissible resistance to be taken into account in epidemiological surveys, and an underappreciated contribution of ICEs to the spread of carbapenem resistance was recently reported (12, 31). The objective of this study was to explore the molecular basis for carbapenem resistance in an XDR clone of P. aeruginosa. We uncovered two GIs carrying MBL-encoding transferable class 1 integrons, inserted into chromosomal sites that can be added to the limited locations for RGP.

## RESULTS

### XDR P. aeruginosa containing both *bla*_IMP-1_and *bla*_DIM-1_.

Four P. aeruginosa strains originating from a collection of MDR clinical Gram-negative bacteria sampled in Ghana in 2015 (32) were selected for further studies due to their carbapenem-resistant phenotype. They were isolated from four different patients located in different wards at the same hospital during a 6-week time span (see Table S1 in the supplemental material). Antimicrobial susceptibility testing demonstrated resistance to agents from all categories of antipseudomonal antimicrobials except polymyxins, with some MIC values differing slightly between the strains (Table 1). According to standard criteria (33), the four strains were all categorized as XDR.

**TABLE 1:** Antimicrobial susceptibility profiles of extensively drug-resistant P. aeruginosa strains

Strain identifier	MIC (mg/liter)^*a*^											
	TZP	CAZ	C/T	CZA	ATM	MEM	IMI	GEN	TOB	AMK	CIP	CST
97	64	>128	>16	>16	16	8	>32	16	128	32	>16	1
130	128	>128	>16	>16	16	8	>32	32	64	32	>16	2
140	>128	>128	>16	>16	16	16	>32	32	>128	64	>16	2
142	>128	>128	>16	>16	16	8	>32	32	128	64	>16	2

a As determined by broth microdilution (Sensititre) and interpreted using EUCAST (67, 68). TZP, piperacillin-tazobactam; CAZ, ceftazidime; C/T, ceftolozane-tazobactam; CZA, ceftazidime-avibactam; ATM, aztreonam; MEM, meropenem; IMI, imipenem; GEN, gentamicin; TOB, tobramycin; AMK, amikacin; CIP, ciprofloxacin; CST, colistin.

Whole-genome sequencing analyses of the four selected strains (Table 2) substantiated the phenotypic findings. All four strains encoded two carbapenemases (DIM-1 and IMP-1) revealing 100% identity to the corresponding prototypes, although three synonymous mutations were observed in *bla*_IMP-1_. Additionally, they encoded two extended-spectrum-β-lactamases, OXA-10 and OXA-129, the latter being most closely related to OXA-5, which is known as a narrow-spectrum β-lactamase (34). Moreover, the strains contained mutations in GyrA (T83I) and ParC (S87L) that are typically involved in high-level fluoroquinolone resistance and are nearly universally associated with P. aeruginosa high-risk clones (16). Other acquired resistance genes included *aadA1*, *aph(3′)-IIb*, *aacA4*, *crpP*, *catB7*, *arr2*, *sul1*, and *dfrB5*; in addition, *aadA6* and *qnrVC1* were present only in strains 97 and 140.

**TABLE 2:** WGS data analyses of the XDR P. aeruginosa strains

Strain identifier	WGS data		GenBank accession no.	GenBank assembly accession no.	Genes encoding acquired resistance to:						
	No. of contigs	Coverage (×)			β-lactams	Aminoglycosides	Fluoroquinolones	Phenicols	Rifampin	Sulphonamide	Trimethoprim
97	1	30	NZ_CP031449.2	GCA_002411865.3	*bla*_OXA-10_, *bla*_OXA-129_, *bla*_DIM-1_, *bla*_IMP-1_	*aadA1*, *aph(3′)-IIb*, *aacA4*, *aadA6*	*crpP*, *qnrVC1*	*catB*7	*arr*-*2*	*sul1*	*dfrB*5
130	274	98	NXHP00000000.1	GCA_002411845.1	*bla*_OXA-10_, *bla*_OXA-129_, *bla*_DIM-1_, *bla*_IMP-1_	*aadA1*, *aph(3′)-IIb*, *aacA4*	*crpP*	*catB*7	*arr*-*2*	*sul1*	*dfrB*5
140	285	111	NXHO00000000.1	GCA_002411815.1	*bla*_OXA-10_, *bla*_OXA-129_, *bla*_DIM-1_, *bla*_IMP-1_	*aadA1*, *aph(3′)-Ilb*, *aacA4*, *aadA6*	*crpP*, *qnrVC1*	*catB*7	*arr*-*2*	*sul1*	*dfrB*5
142	378	54	NXHN00000000.1	GCA_002411785.1	*bla*_OXA-10_, *bla*_OXA-129_, *bla*_DIM-1_, *bla*_IMP-1_	*aadA1*, *aph(3′)-IIb*, *aacA4*	*crpP*	*catB*7	*arr*-*2*	*sul1*	*dfrB*5

### Independent acquisition of the carbapenemase-encoding determinants.

An overall low prevalence of *bla*_DIM-1_-positive (*n *= 4) and *bla*_IMP-1_-positive (*n *= 15) P. aeruginosa genomes (listed in Table S2 in the supplemental material) were found by protein BLAST search in the NCBI database. Of particular interest, genome 1334/14 (*bla*_DIM-1_) from Poland belonged to the same sequence type determined for the four genomes in our study (ST234), although it was shown to carry *bla*_DIM-1_ on a genetic structure not present in the Ghanaian strains (35). The other genomes with *bla*_DIM-1_ or *bla*_IMP-1_ were assigned to 12 different STs, with none encoding both genes, implicating dissemination by horizontal transfer rather than by clonal spread.

**FIG 1:**
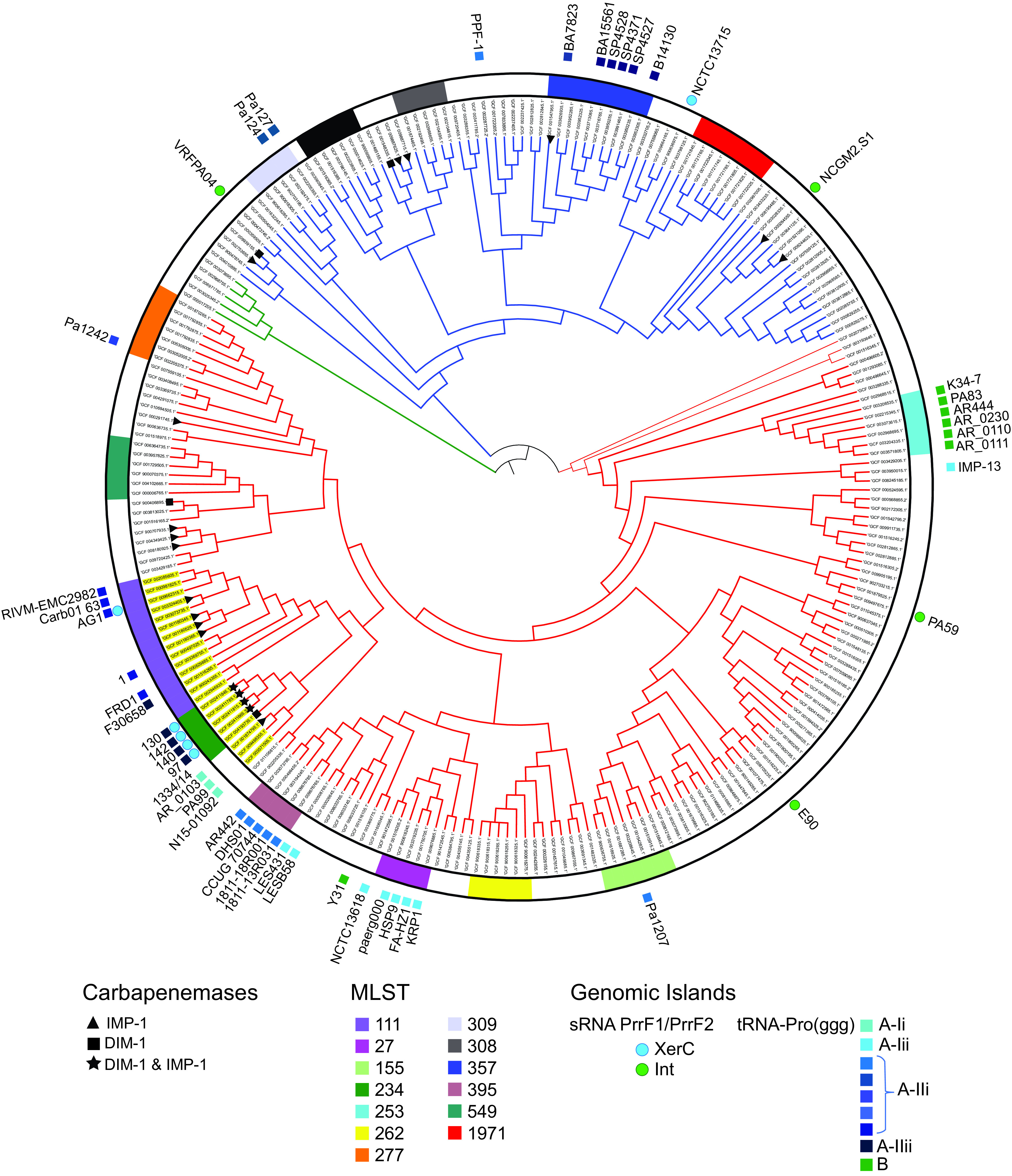
Core genome phylogeny of P. aeruginosa. The tree is based on closed genomes with the addition of all *bla*_IMP-1_ and *bla*_DIM-1_ whole-genome sequence data (*n *= 243) available in GenBank. Phylogenetic main groups 1, 2, and 3 (red, blue, and green, respectively) and members of the sequence type 111 (ST111)-containing clade are highlighted (yellow). For each genome, assembly number, multilocus sequence type (MLST, innermost ring; color codes are shown for STs assigned to ≥5 genomes), and presence of *bla*_IMP-1_ (triangle), *bla*_DIM-1_ (black square), or both (star) are shown. In the outer ring, genomic islands (GIs) integrated into small RNA (sRNA) PrrF1/PrrF2 (circles) or tRNA-Pro (squares) are shown with color codes referring to the type of recombinase/integrases (XerD or Int) or to the *intG* phylogroup, respectively. The tree was midpoint routed and metadata were added using iTOL (https://itol.embl.de/).

P. aeruginosa core genome phylogeny (Fig. 1) confirmed a wide distribution of *bla*_DIM-1_- and *bla*_IMP-1_-encoding strains into various subclades of phylogenetic main groups 1 and 2. The genomes of the four Ghanaian strains formed a cluster, with genomes 1334/14 and AR_0103 (ST964; *bla*_IMP-1_) present in paraphyletic groups. Together with the ST111 genomes, they constitute a phylogenetic clade with a total of 10 strains carrying *bla*_IMP-1_ and/or *bla*_DIM-1_ (Fig. 1). Relatedness addressed by core genome single-nucleotide polymorphisms (SNPs) confirmed clonality between the genomes from our study (25 to 190 SNPs), whereas more distant relations to 1334/14 (∼1,500 SNPs), AR_0103 (∼21,500 SNPs), and AG1 (ST111; ∼25,500 SNPs), representing the other defined paraphyletic groups in the ST111 clade, were confirmed. Taken together, these results point to multiple independent acquisitions of *bla*_DIM-1_ and *bla*_IMP-1_ followed by clonal expansions, also within the ST111 clade.

### Tn*402*-type class 1 integrons containing *bla*_DIM-1_ or *bla*_IMP-1_.

The *bla*_IMP-1_- and *bla*_DIM-1_-containing contigs were identical for all four genomes (2,659 and 3,093 nt, respectively) and gave limited information regarding genetic context. To obtain further insight into the acquisition of these MBL-encoding genes, one of the genomes was completed by Oxford Nanopore Technologies (ONT) long-read sequencing. The genome of strain 97 had a chromosome size of 6,925,889 bp, a GC content of 65.89%, and contained no plasmids.

**FIG 2:**
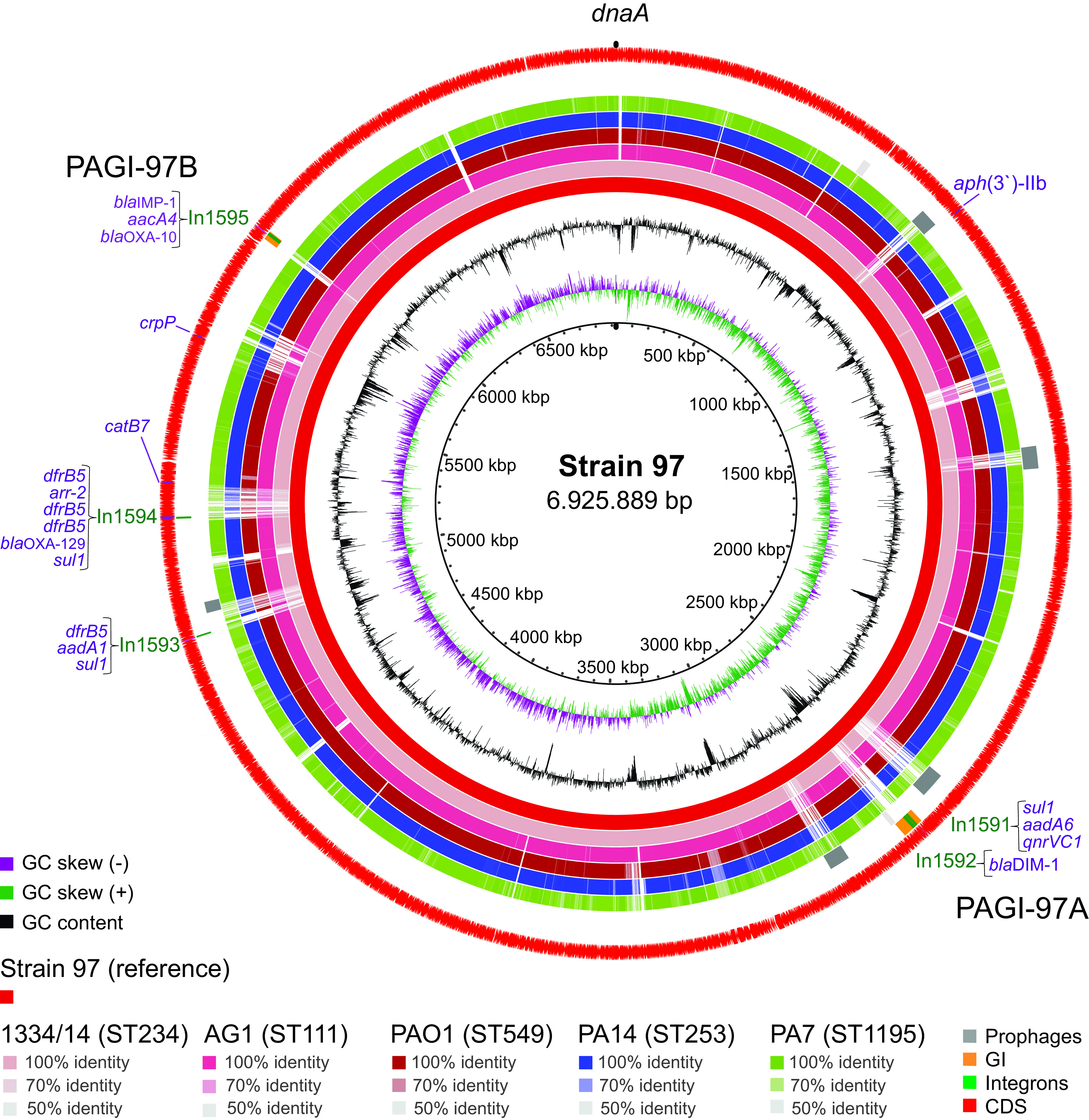
Chromosome of P. aeruginosa strain 97 used as reference for BLAST alignments. In the circularized chromosome, CG skew and GC content are indicated (two innermost circles), as well as annotated coding sequences (CDS; red arrows, outermost circle) with acquired resistance genes (lilac). Arcs showing GIs (orange), integrons (green), intact (dark gray), or questionable (light gray) prophages are shown in the next circles. In the BLAST comparisons with the selected genomes from phylogenetic main groups 1 (red), 2 (blue), and 3 (green), DNA identity (50 to 100%) to the reference is visualized by the given color codes, with missing regions appearing as white. The map was constructed using BRIG software (http://brig.sourceforge.net/).

A majority of the resistance genes were carried by five novel class 1 integrons (see Table S3 in the supplemental material) identified at different positions of the chromosome (Fig. 2). Of particular interest were In1592 (8,945 bp) and In1595 (8,592 bp), which contained *bla*_DIM-1_ and *bla*_IMP-1_, respectively. Both carried a conserved *tni* module (92% identical to each other) encoding complete arrays of transposition genes (*tniR*, *tniQ*, *tniB*, and *tniA*) originally detected in Tn*402*-type integrons (36, 37). Moreover, In1595 was delineated by 25-bp inverted repeats (IRs), 100% identical to the IRs reported for the complete versions of Tn*402*-related elements (36). The presence of 5-bp DRs (TCAAA) bracketing the IRs strongly indicate a small target duplication associated with a transposition event. Single copies of the conserved 25-bp sequence were detected in the 3′ or 5′ ends of the four additional integrons (Table S3).

### Two chromosomally integrated carbapenemase-encoding genomic islands.

**FIG 3:**
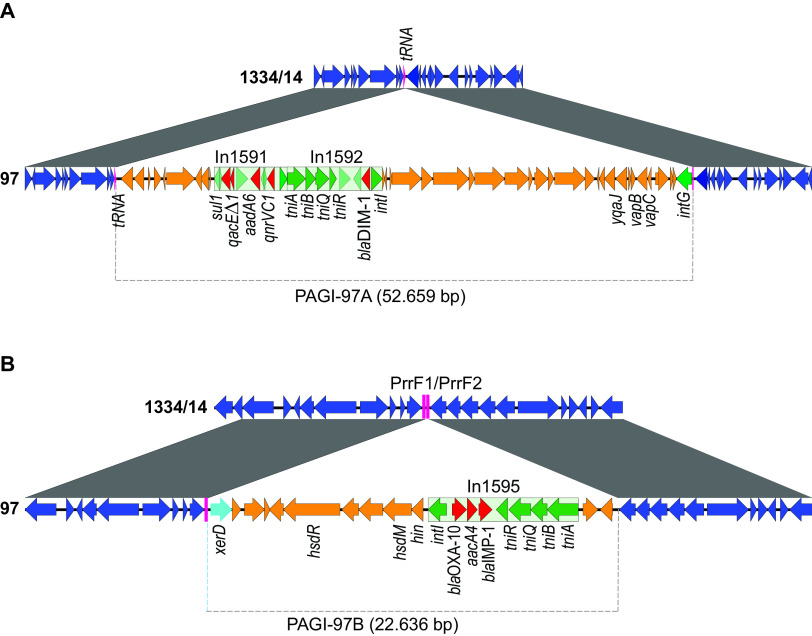
Carbapenemase-encoding GIs (PAGI-97A and PAGI-97B) inserted into the chromosome of P. aeruginosa strain 97. Integrated DNA containing (A) *bla*_DIM-1_ and (B) *bla*_IMP-1_ were delineated (gray dotted line) between regions of 100% identity (gray) by pairwise chromosome alignments with strain 1334/14 (GenBank accession number NZ_CP035739.1). Open reading frames are indicated by arrows pointing in the direction of transcription, with genes belonging to the backbone (blue) and to the GIs (orange), resistance (red), tRNA genes (magenta), *intG* (green), and *xerD* (turquoise) highlighted. The integron (light green box) backbone genes are marked in green and direct repeats (DRs) are indicated (magenta box).

To investigate their insertion sites as well as genetic content, the *bla*_DIM-1_- and *bla*_IMP-1_-containing genetic elements were delineated by sequence alignments. Among the completed genomes phylogenetically related to strain 97, 1334/14 was selected for pairwise alignments due to the high DNA identity in the flanking regions of both In1592 and In1595, as revealed by BLAST analyses. Flanked by 100% identical DNA, two distinct insertions were identified in strain 97 compared to 1334/14, PAGI-97A (52,659 bp) and PAGI-97B (22,636 bp) (Fig. 3). As is characteristic for GIs, their GC contents of 54.7% (PAGI-97A) and 57.3% (PAGI-97B) were markedly different from the genome average, and GC peaks corresponding to the two GIs (positions 2,622,067 to 2,674,677 and 5,905,810 to 5,928,446) could be identified in the chromosome map (Fig. 2). We did not recognize any conjugation genes among their annotated coding sequences (CDS), and no origin of transfer was identified in their DNA sequences, showing that these GIs do not belong to the ICEs.

NCBI BLAST alignments using the chromosome of strain 97 as a reference (Fig. 2) revealed additional sections with no DNA identity to 1334/14. These can be interpreted as RGP, known to cluster at limited chromosomal loci in P. aeruginosa (25). Other putative RGP were identified when comparing to less related genomes from phylogenetic main groups 1 (AG1; PAO1), 2 (PA14), and 3 (PA7). Several of these regions corresponded to prophages, one of which was situated in the same highly variable region of the chromosome as PAGI-97A. BLAST comparisons showed high DNA identity of the region immediately downstream of PAGI-97A among the ST234 genomes available in the PATRIC database (*n *= 11), suggesting a recent acquisition of this island. PAGI-97B, on the other hand, was embedded in a more conserved region of the chromosome.

### Site-specific integration of PAGI-97A into tRNA-Pro(ggg).

Bacterial tRNA genes have commonly been recognized as target sites for chromosomal integration of various genetic elements by site-specific recombinases (38). Here, the tRNA-Pro(ggg) gene locus, which was present in a single copy, was identified as the insertion point for PAGI-97A (Fig. 3A). In accordance with previously described tRNA integrations (39), we detected a 3′-end duplication of the tRNA gene, resulting in a DR of 49 bp bracketing the GI. The GI encoded a cognate integrase, IntG, containing a C-terminal P4-type site-specific integrase of the tyrosine recombinase family, as recognized by NCBI Conserved Domain Database (CDD), in its 3′ border. Additionally, the GI carried resistance genes contained by In1592 (*bla*_DIM-1_) and In1591 (*sul1*, *qnrVC1*, *qacE*Δ1, and *aadA6*), genes encoding a toxin-antitoxin system (*vapBC*) and a viral recombinase (*yqaJ*), insertion sequence (IS) elements, and, additionally, genes encoding mostly unknown hypothetical proteins, for a total of ∼50 CDS.

NCBI BLAST search showed that this GI was unique to the four genomes in our study. However, an examination of the completed P. aeruginosa genomes (*n *= 226) available in the NCBI database, identified chromosomal elements with structural features similar to PAGI-97A, but differing in genetic cargo, in a total of 41 strains (listed in Table S2 in the supplemental material). Specific for this group of GIs was that they carried *intG* in the 3′ terminus and were delineated by the 49-bp DR identified at different distances (21.2 to 102.9 kbp) from tRNA-Pro(ggg). In the core genome tree, they were found in a variety of clades in phylogenetic main groups 1 and 2 (Fig. 1), implicating horizontal spread.

**FIG 4:**
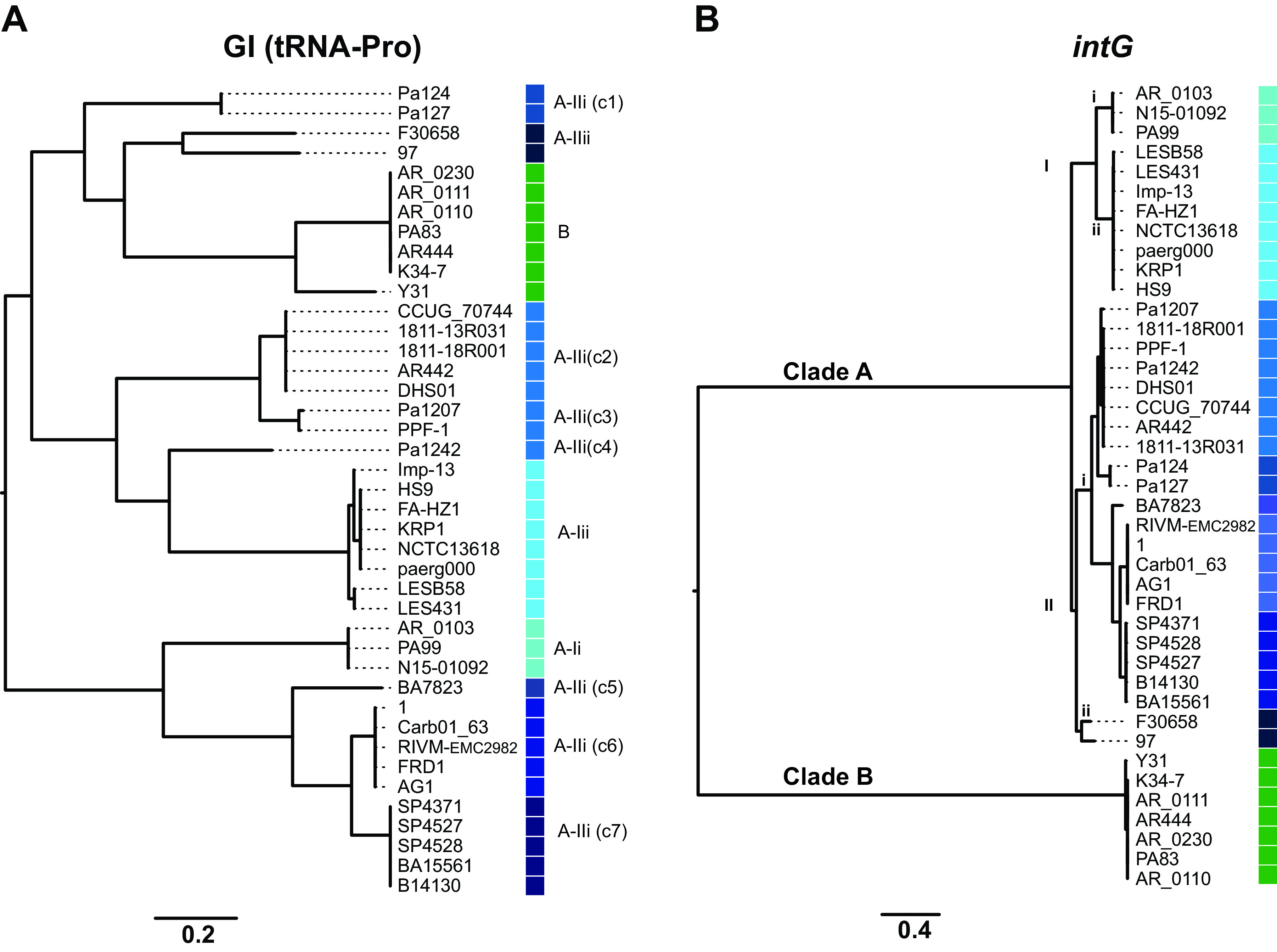
Phylogenetic analyses of GIs integrated into tRNA-Pro(ggg) in P. aeruginosa. Tree based on nucleotide sequence of the entire GIs (A) or on *intG* B) from the corresponding GIs from each strain. Phylogroups of *intG*, including clade A with clusters (I and II) and subclusters (i and ii) and clade B, are indicated by color codes, and clusters of GIs (c1 to c7) within the A-IIi group are marked. Individual scale bars represent genetic distances.

To explore the dissemination and relatedness of these GIs with regard to genetic cargo as well as their cognate integrases, we constructed phylogenetic trees based on their complete nucleotide sequence or on *intG* only (Fig. 4). The tree revealed an overall high level of diversity of the GIs (Fig. 4A), with several distinct clusters as well as unique ones, like PAGI-97A from strain 97. GIs from the same clusters were mainly present in phylogenetically highly related genomes (indicated in Fig. 1) and might be regarded as clonally disseminated homologs. Interestingly, within some of these genome clusters, which also belong to the same ST, strains where the specific GI is absent can be observed. This implicates recent gain or loss of the GI and therefore integration/excision activities.

Additional comparisons of the GIs were conducted through phylogeny of their cognate integrase (Fig. 4B), defining clade A with clusters (I and II) and subclusters (i and ii), and clade B. Highly related GIs contained *intG* from either clade B or one of the clade A subclusters, and further subgrouping of A-Iii correlated exactly to GI subclusters c1 to c7 (Fig. 4A). However, the overall relatedness of the GIs, including that of the c1 to c7 clusters, did not correlate with the *intG* phylogeny, indicating independent evolution and clonal relatedness. Moreover, GIs from some of the groups (B, A-Iii, and A-IIi-c3) were found in unrelated genomes, which suggests that these were independently acquired.

The DNA identity was ≥99% within clade B and the clade A subclusters of *intG* and ≥92.3% between the clade A subclusters. Surprisingly, *intG* from clades A and B displayed no detectable DNA homology in BLAST alignment. At the amino acid level, they revealed 40% identity and, importantly, shared the C-terminal P4-like integrase domain. These findings disclose additional complexity of the evolutionary relationship of this group of GIs.

Taken together, these GIs share a mechanism for site-specific integration that is mediated by their inherent integrase, which renders tRNA-Pro(ggg) as their specific recognition site and thereby a location for genome plasticity in P. aeruginosa.

### The conserved sRNA PrrF1/PrrF2 loci as a genomic integration site for PAGI-97B.

**FIG 5:**
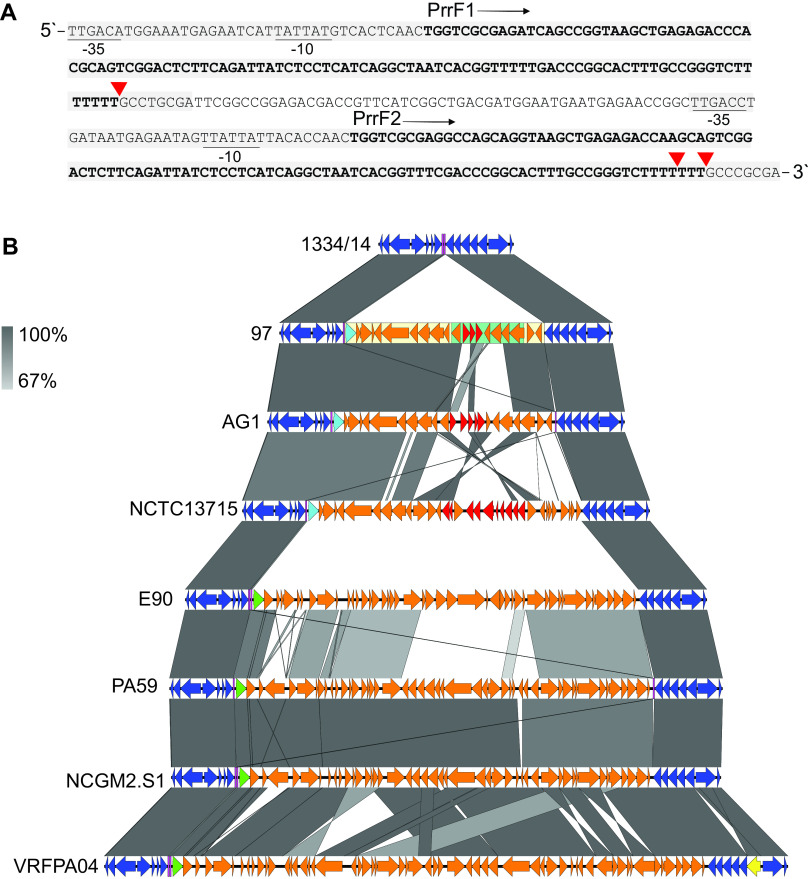
Genomic integrations into the sRNA PrrF1/PrrF2 loci of P. aeruginosa. (A) DNA sequence encoding the two regulatory small RNAs (PrrF1 and PrrF2; bold) with promoter elements (−35 and −10) and DRs (159/156 bp; boxed in gray) indicated. Red triangles mark insertion sites of GIs. (B) Alignments of GIs (*n *= 7) detected in the indicated strains, where the chromosomal insertion site is represented by strain 1334/14. Open reading frames are indicated by arrows pointing in the direction of transcription, with genes belonging to the chromosomal backbone (blue), genomic islands (orange), resistance (red), insertion sequence (IS) element (yellow), and the *xerD* (turquoise) or *int* (green) recombinase/integrase-encoding genes highlighted. PAGI-97B (light yellow), In1595 (green), and DRs (magenta) are boxed, and a gray scale for DNA identity (67 to 100%) is shown.

In its 5′ border (Fig. 3B), a XerC/D recombinase containing a C-terminal DNA breaking-rejoining catalytic domain, known to be involved in unidirectional site-specific DNA recombination, was recognized by CDD search. Additional genetic content included In1595 (*bla*_OXA-10_, *aacA4*, and *bla*_IMP-1_), a type I restriction system (*hsdR* and *hsdM*), and hypothetical proteins with unknown functions, for a total of ∼20 CDS. The GI was integrated exactly downstream of the PrrF1-encoding gene of the intergenic PrrF1/PrrF2 locus (Fig. 5A), which is involved in iron-dependent homeostasis (40). The locus encodes the two independently expressed small RNA (sRNA; 92% identical) and forms imperfect DRs of 159/156 bp separated by 53 bp. In strain 97, the 5′-end part of the locus, including PrrF2, was missing due to the insertion of PAGI-97B.

Although NCBI BLAST search confirmed that the PrrF1/PrrF2 locus is highly conserved in P. aeruginosa, integrations (∼20 to 50 kb) were detected in six additional, phylogenetically unrelated genomes. DNA alignments that included the wild-type locus represented by 1334/14 enabled a precise detection of insertion point and delineation and comparison of these GIs (Fig. 5B). Two more genomes had GIs (AG1, 21,154 bp; NCTC13715, 31,074 bp) that carried *xerD* in the 5′ end, inserted into the same site (Fig. 5B). They exhibited extensive sequence identity to PAGI-97B, except for the integron gene cassette regions. AG1 belongs to the ST111 clade, whereas NCTC13715 is phylogenetically unrelated to strain 97.

It is noteworthy that in AG1, the complete 5′-end part of the PrrF1/PrrF2 locus was detected downstream of the insertion. This could implicate that the GIs originally were integrated between PrrF1 and PrrF2, and the downstream part of the locus was lost thereafter in strain 97 and NCTC13715. It is also of interest that truncations of the 5′-end part of the locus were discovered at a position identical to that of the GI integration site in the genomes of Pa127 (GenBank assembly accession number GCF_002205355.1) and ATCC 27853 (accession number GCF_001687285.1). This might be explained by loss of integrated DNA by homologous recombination between the two DRs. In PA59, DNA (47,568 bp) was inserted in the same manner as in AG1, although this GI carried *int* in the 5′ end and had no sequence identity to the ones described above. The alignments showed, however, extensive DNA identity, including that of the 5′-end *int*, to the corresponding insertions in the phylogenetically unrelated genomes of E90 (43,995 bp), NCGM2.S1 (47,189 bp), and VRFFP04 (60,277 bp). Adding to the complexity, two of these GIs were inserted into a site almost identical to PAGI-97B, exactly downstream of PrrF2, truncating the last 8 bp of the second DR of this locus, while in VRFFP04, the GI was inserted upstream of the last codon of PrrF2 (Fig. 5A).

In summary, we have identified two phylogenetically unrelated groups of GIs, encompassing different enzymes anticipated to be involved in their integration into the same highly conserved intergenic locus, which forms imperfect DRs. The locus was disrupted downstream of either PrrF1 or PrrF2, keeping one or both of these 90% identical regulatory sRNA genes intact.

## DISCUSSION

The global dissemination of carbapenemase-producing P. aeruginosa is facilitated by an interplay between mobile genetic elements and successful clones (12, 31, 41). Our study identified dissemination of an XDR ST234 clone phylogenetically related to the ST111 high-risk clone, having two horizontally acquired carbapenemase-encoding determinants, *bla*_DIM-1_ and *bla*_IMP-1_, embedded in the chromosome. DIM is a relatively rare MBL and was initially described in a clinical isolate of Pseudomonas stutzeri in The Netherlands (42), and thereafter in clinical isolates of Pseudomonadaceae, Enterobacteriaceae, Burkholderiaceae, and Comamonadaceae in Sierra Leone (43), and Pseudomonas putida in China (44). Isolation of an additional clinical P. stutzeri strain in India with *bla*_DIM-1_ contained in a genetic environment different from that of the index strain led to the suggestion of an unidentified environmental source of this MBL (45). Coproduction of more than one MBL has recently been reported in clinal P. aeruginosa strains in Iran (46) but is not commonly found.

Unlike the class 1 integrons that are generally associated with carbapenemase-encoding genes in P. aeruginosa (9, 13, 41, 47), both *bla*_DIM-1_ and *bla*_IMP-1_ were carried by integrons possessing complete *tni* modules with boundaries defined by inverted repeats, features typical for Tn*402*-like class 1 integrons (36). For In1595, the observed 5-bp target duplication indicates a recent acquisition within PAGI-97B in strain 97, which is also supported by the absence of a corresponding integron in a homologous GI in P. aeruginosa AG1. The four *tni* genes enable active transposition to *res* sites of Tn*21* subfamily transposons but also to resolution sites found on plasmids, and are implicated in integron dissemination (10, 48, 49). Integrons of this type, also termed “preclinical class 1 integrons,” are suggested to be key evolutionary intermediates still circulating in the environment and capable of acquiring gene cassettes (50). Variants denoted “unusual” class 1 integrons, due to the lack of the 3′ conserved sequence (*qacE*Δ1-*sul1*) found in the vast majority of class 1 integrons in clinically relevant bacteria, have been reported in clinical P. aeruginosa and P. putida isolates, where they contained *bla*_VIM-2_ and *aacA4* (51, 52).

GIs play a key role in prokaryotic genome plasticity and are recognized in P. aeruginosa as a major factor in acquisition and dissemination of multiple antibiotic resistance genes (31, 35, 53–55), as well as in virulence traits (56–58). Our bioinformatic analyses revealed that both chromosomal regions containing the carbapenemase-encoding integrons exhibited GI features (57, 59, 60), including size, decreased GC content, presence of integrase/recombinase genes, and, for PAGI-97A, integration into a tRNA gene and flanking by DRs. In line with our findings, GIs usually carry genes encoding hypothetical proteins whose functions are unknown (30, 61). The two GIs were uniquely detected in the Ghanaian clone and are absent in highly related genomes, including 1334/14, which belongs to the same ST234 lineage, and have 100% protein identity within 5,700 CDS (35), which argues for a recent acquisition by horizontal transfer from an unknown donor source.

Mobile genetic elements that integrate into the host chromosomes either carry their own DNA integration machineries or exploit machineries already existing in the bacterial host (10). Integrases associated with GIs form a separate clade within the tyrosine recombinase family and are frequently located at the 5′ border of the GI, close to the tRNA locus or the respective attachment site (59, 60, 62). In P. aeruginosa, GI integrations have been detected in defined tRNA genes, including tRNA-Gly, tRNA-Thr, and tRNA-Phe, but also in structural genes such as *hrpB* (53, 57, 58, 63). Our study revealed tRNA-Pro(ggg) as an additional site for specific GI integrations. Although these GIs belonged to several phylogroups based on genetic content, they all encoded P4-type integrases of the tyrosine recombinase family in their 3′ rather than 5′ border, and were bracketed by 49-bp DRs resulting from 3′-end tRNA-Pro(ggg) target duplications. With these specific features in common, they might be regarded as a distinct family of GIs with specificity determined by their common P4 integrase. In line with our observations of phylogenetically related genomes revealing presence or absence for a specific GI in this site is the reported instability due to the flanking DRs, which are often homologous to phage attachment sites and promote integration and excision (64, 65).

Three genetically related XerD-encoding GIs were inserted into the highly conserved sRNA PrrF1/PrrF2 locus. This intergenic locus encodes tandem sRNAs, which are >95% identical to each other and independently expressed, with an overlapping central function in the regulation of iron homeostasis and pathogenesis in P. aeruginosa (40, 66). Despite the GIs, their important regulatory functions might be restored due to the insertion sites directly downstream of the PrrF1 gene. In the AG1 chromosome, the complete 3′ part of the locus is present downstream of the GI, which positions the two imperfect DRs that constitute the PrrF1 and PrrF2 genes at the GI boundaries, where they might facilitate the Xer-mediated excision and integration. It can be speculated that truncation after integration explain the absence of the 3′ part of the locus downstream of the GIs in strains 97 and NTC13715, and that the DRs themselves might be of importance for the integration activity. However, the finding of an unrelated group of GIs inserted into the locus at additional positions, and where XerD is replaced by a different integrase, add complexity that needs to be resolved by future studies.

### Main conclusions.

The presence of class 1 integrons with complete *tni* mobilization modules located on GIs in high-risk P. aeruginosa strains are of great clinical concern with regard to the dissemination of carbapenem resistance. Moreover, the molecular characterization of the two carbapenemase-encoding GIs and their chromosomal integration sites has added two novel locations for RGP in P. aeruginosa and enabled the identification of additional GIs with integration specificities toward these sites. Finally, our findings emphasize the importance of GIs in the dissemination of antibiotic resistance determinants in P. aeruginosa.

## MATERIALS AND METHODS

Approval for this study was given by the Joint Committee on Human Research, Publications and Ethics, School of Medical Sciences, Kwame Nkrumah University of Technology, the Ethics Committee of Komfo Anokye Teaching Hospital (reference no. CHRPE/AP/015/15) in Kumasi Ghana, and the Biomedical Research Ethics Committee of the University of KwaZulu-Natal, South Africa (reference no. BE 494/14).

### Bacterial characterization and selection.

Relevant patient data and sources of specimens for the four carbapenem-resistant P. aeruginosa strains selected for this study are shown in Table S1 in the supplemental material. The strains were isolated from four patients in three different wards and originated from a collection of 200 clinical, nonduplicate, Gram-negative bacterial isolates sampled from inpatients at the Komfo Anokye Teaching Hospital in Ghana between February and August 2015 (32). Isolates were identified by the Vitek 2 (bioMérieux) automated system and confirmed by matrix-assisted laser desorption ionization–time of flight mass spectrometry (MALDI-TOF MS; Bruker Daltonic Gmbh). Antimicrobial susceptibility testing was conducted by broth microdilution (Sensititre, Trek Diagnostic Systems) and interpreted according to European Committee on Antimicrobial Susceptibility Testing (EUCAST) guidelines (67, 68).

### DNA sequencing.

For Illumina sequencing, DNA was extracted using the PureLink Microbiome DNA purification kit according to the instructions of the manufacturer (Invitrogen). DNA libraries were generated using the Nextera kit and sequenced on an Illumina NextSeq 550 system at the Genomics Support Centre Tromsø (Norway). For long-read sequencing by MinION (Oxford Nanopore Technologies; ONT), genomic DNA was purified using the Qiagen Genomic-tip 100/G kit, and a DNA library prepared using a rapid kit (SQK-RAD001) and sequenced on a R9.4 flow cell (FLO-MIN106) supplied by ONT, all according to the manufacturer’s instructions.

### DNA sequence analysis.

Illumina raw sequences from all four genomes were quality trimmed and adapters removed with Trimmomatic v0.35 (69) in paired-end mode, followed by SPAdes v3.11.0 (70) assembly with quality scores assessed by QUAST v4.6.0 (71). The fast5 reads resulting from the MinION sequencing of one selected genome were base-called using Albacore v2.1.7 (https://nanoporetech.com), and adapters were trimmed off by Porechop v0.2.3 (https://github.com/rrwick/Porechop). Corrected nanopore fastq files were assembled along with corresponding Illumina reads by Unicycler v0.4.6, selecting the normal mode (72). All of the assembled genomes were annotated with Prokka v1.14.0 (73). Each genome was classified taxonomically based on both the reads and contigs by Kraken v1.1 (74). Multilocus sequence type (MLST) was determined by MLST software (https://github.com/tseemann/mlst). Resistance genes were identified by abricate-0.8 (https://github.com/tseemann/abricate) and the NCBI Bacterial Antimicrobial Resistance Reference Gene Database (BioProject accession number PRJNA313047). Unknown functional genes were scanned for conserved protein domains by NCBI Conserved Domain Database (CDD) search (https://www.ncbi.nlm.nih.gov/Structure/cdd/wrpsb.cgi). oriTfinder (https://bioinfo-mml.sjtu.edu.cn/oriTfinder) was employed to identify origin of transfer in DNA sequences of mobile genetic elements.

Global phylogeny was constructed using Parsnp v1.2 with the “-c” flag enabled, using strain 97 as the reference on all the completed P. aeruginosa genomes, together with *bla*_IMP-1_ and *bla*_DIM-1_-containing genomes (*n *= 243, listed in Table S2 in the supplemental material). The genome assemblies used in the phylogenetic and comparative analyses, including corresponding metadata, were downloaded (16 April 2020) from the Pathosystems Resource Integration Center (PATRIC) database (https://www.patricbrc.org/). FigTree (http://tree.bio.ed.ac.uk/software/figtree/) was used to visualize and curate the phylogenetic trees, and metadata were coupled to specific genomes using iTOL (https://itol.embl.de/).

Integrons were curated by the Integral database (http://integrall.bio.ua.pt/), and phage regions were identified by PHASTER (https://phaster.ca/). Single-nucleotide polymorphisms (SNPs) between the genomes were predicted using Snippy v4.4.3 (https://github.com/tseemann/snippy). BLAST results were visualized by the Artemis Comparison Tool (https://www.sanger.ac.uk/tool/artemis-comparison-tool-act/) or by BLAST Ring Image Generator (BRIG), where default values, except for shading (false), were used for all parameters (75).

### Accession number(s).

The raw read sequences and the assembled whole-genome contigs for strains 97, 130, 140, and 142 have been deposited in GenBank under BioProject accession number PRJNA411997. The closed genome of strain 97 has accession number NZ_CP031449.2.

## References

[1] Lyczak JB, Cannon CL, Pier GB. 2000. Establishment of *Pseudomonas aeruginosa* infection: lessons from a versatile opportunist. Microbes Infect 2:1051–1060. 10.1016/S1286-4579(00)01259-4. 10967285

[2] Silby MW, Winstanley C, Godfrey SAC, Levy SB, Jackson RW. 2011. *Pseudomonas* genomes: diverse and adaptable. FEMS Microbiol Rev 35:652–680. 10.1111/j.1574-6976.2011.00269.x. 21361996

[3] Palavutitotai N, Jitmuang A, Tongsai S, Kiratisin P, Angkasekwinai N. 2018. Epidemiology and risk factors of extensively drug-resistant *Pseudomonas aeruginosa* infections. PLoS One 13:e0193431. 10.1371/journal.pone.0193431. 29470531 PMC5823452

[4] Codjoe F, Donkor E. 2018. Carbapenem resistance: a review. Med Sci 6:1–28. 10.3390/medsci6010001. PMC5872158 29267233

[5] Bush K, Jacoby GA. 2010. Updated functional classification of β-lactamases. Antimicrob Agents Chemother 54:969–976. 10.1128/AAC.01009-09. 19995920 PMC2825993

[6] Botelho J, Grosso F, Peixe L. 2019. Antibiotic resistance in *Pseudomonas aeruginosa*—mechanisms, epidemiology and evolution. Drug Resist Updat 44:26–47. 10.1016/j.drup.2019.07.002. 31492517

[7] Hong DJ, Bae IK, Jang I-H, Jeong SH, Kang H-K, Lee K. 2015. Epidemiology and characteristics of metallo-β-lactamase-producing *Pseudomonas aeruginosa*. Infect Chemother 47:81–97. 10.3947/ic.2015.47.2.81. 26157586 PMC4495280

[8] Castanheira M, Deshpande LM, Costello A, Davies TA, Jones RN. 2014. Epidemiology and carbapenem resistance mechanisms of carbapenem-non-susceptible *Pseudomonas aeruginosa* collected during 2009–11 in 14 European and Mediterranean countries. J Antimicrob Chemother 69:1804–1814. 10.1093/jac/dku048. 24603963

[9] van der Zee A, Kraak WB, Burggraaf A, Goessens WHF, Pirovano W, Ossewaarde JM, Tommassen J. 2018. Spread of carbapenem resistance by transposition and conjugation among *Pseudomonas aeruginosa*. Front Microbiol 9:2057. 10.3389/fmicb.2018.02057. 30233535 PMC6133989

[10] Partridge SR, Kwong SM, Firth N, Jensen SO. 2018. Mobile genetic elements associated with antimicrobial resistance. Clin Microbiol Rev 31:e00088-17. 10.1128/CMR.00088-17. 30068738 PMC6148190

[11] Mazel D. 2006. Integrons: agents of bacterial evolution. Nat Rev Microbiol 4:608–620. 10.1038/nrmicro1462. 16845431

[12] Botelho J, Roberts AP, León-Sampedro R, Grosso F, Peixe L. 2018. Carbapenemases on the move: it’s good to be on ICEs. Mob DNA 9:37. 10.1186/s13100-018-0141-4. 30574213 PMC6299553

[13] Diene SM, Rolain JM. 2014. Carbapenemase genes and genetic platforms in Gram-negative bacilli: *Enterobacteriaceae*, *Pseudomonas* and *Acinetobacter* species. Clin Microbiol Infect 20:831–838. 10.1111/1469-0691.12655. 24766097

[14] Curran B, Jonas D, Grundmann H, Pitt T, Dowson CG. 2004. Development of a multilocus sequence typing scheme for the opportunistic pathogen *Pseudomonas aeruginosa*. J Clin Microbiol 42:5644–5649. 10.1128/JCM.42.12.5644-5649.2004. 15583294 PMC535286

[15] Oliver A. 2017. Epidemiology and carbapenem resistance mechanisms in *Pseudomonas aeruginosa*: role of high-risk clones in multidrug resistance. Enferm Infecc Microbiol Clin 35:137–138. 10.1016/j.eimce.2017.02.006. 28161004

[16] López-Causapé C, Cabot G, del Barrio-Tofiño E, Oliver A. 2018. The versatile mutational resistome of *Pseudomonas aeruginosa*. Front Microbiol 9:685. 10.3389/fmicb.2018.00685. 29681898 PMC5897538

[17] Oliver A, Mulet X, López-Causapé C, Juan C. 2015. The increasing threat of *Pseudomonas aeruginosa* high-risk clones. Drug Resist Updat 21–22:41–59. 10.1016/j.drup.2015.08.002. 26304792

[18] del Barrio-Tofiño E, López-Causapé C, Oliver A. 2020. *Pseudomonas aeruginosa* epidemic high-risk clones and their association with horizontally-acquired β-lactamases: 2020 update. Int J Antimicrob Agents 56:106196. 10.1016/j.ijantimicag.2020.106196. 33045347

[19] Freschi L, Vincent AT, Jeukens J, Emond-Rheault J-G, Kukavica-Ibrulj I, Dupont M-J, Charette SJ, Boyle B, Levesque RC. 2019. The *Pseudomonas aeruginosa* pan-genome provides new insights on its population structure, horizontal gene transfer, and pathogenicity. Genome Biol Evol 11:109–120. 10.1093/gbe/evy259. 30496396 PMC6328365

[20] Ozer EA, Nnah E, Didelot X, Whitaker RJ, Hauser AR. 2019. The population structure of *Pseudomonas aeruginosa* is characterized by genetic isolation of *exoU*^+^ and *exoS*^+^ lineages. Genome Biol Evol 11:1780–1796. 10.1093/gbe/evz119. 31173069 PMC6690169

[21] Freschi L, Bertelli C, Jeukens J, Moore MP, Kukavica-Ibrulj I, Emond-Rheault J-G, Hamel J, Fothergill JL, Tucker NP, McClean S, Klockgether J, de Soyza A, Brinkman FSL, Levesque RC, Winstanley C. 2018. Genomic characterisation of an international *Pseudomonas aeruginosa* reference panel indicates that the two major groups draw upon distinct mobile gene pools. FEMS Microbiol Lett 365:1–11. 10.1093/femsle/fny120. 29897457

[22] Fischer S, Klockgether J, Morán Losada P, Chouvarine P, Cramer N, Davenport CF, Dethlefsen S, Dorda M, Goesmann A, Hilker R, Mielke S, Schönfelder T, Suerbaum S, Türk O, Woltemate S, Wiehlmann L, Tümmler B. 2016. Intraclonal genome diversity of the major *Pseudomonas aeruginosa* clones C and PA14. Environ Microbiol Rep 8:227–234. 10.1111/1758-2229.12372. 26711897 PMC4819714

[23] Spencer DH, Kas A, Smith EE, Raymond CK, Sims EH, Hastings M, Burns JL, Kaul R, Olson MV. 2003. Whole-genome sequence variation among multiple isolates of *Pseudomonas aeruginosa*. J Bacteriol 185:1316–1325. 10.1128/JB.185.4.1316-1325.2003. 12562802 PMC142842

[24] Cramer N, Klockgether J, Wrasman K, Schmidt M, Davenport CF, Tümmler B. 2011. Microevolution of the major common *Pseudomonas aeruginosa* clones C and PA14 in cystic fibrosis lungs. Environ Microbiol 13:1690–1704. 10.1111/j.1462-2920.2011.02483.x. 21492363

[25] Kung VL, Ozer EA, Hauser AR. 2010. The accessory genome of *Pseudomonas aeruginosa*. Microbiol Mol Biol Rev 74:621–641. 10.1128/MMBR.00027-10. 21119020 PMC3008168

[26] Mathee K, Narasimhan G, Valdes C, Qiu X, Matewish JM, Koehrsen M, Rokas A, Yandava CN, Engels R, Zeng E, Olavarietta R, Doud M, Smith RS, Montgomery P, White JR, Godfrey PA, Kodira C, Birren B, Galagan JE, Lory S. 2008. Dynamics of *Pseudomonas aeruginosa* genome evolution. Proc Natl Acad Sci U S A 105:3100–3105. 10.1073/pnas.0711982105. 18287045 PMC2268591

[27] Klockgether J, Cramer N, Wiehlmann L, Davenport CF, Tümmler B. 2011. *Pseudomonas aeruginosa* genomic structure and diversity. Front Microbiol 2:150. 10.3389/fmicb.2011.00150. 21808635 PMC3139241

[28] Sood U, Hira P, Kumar R, Bajaj A, Rao DLN, et al. 2019. Comparative genomic analyses reveal core-genome-wide genes under positive selection and major regulatory hubs in outlier strains of *Pseudomonas aeruginosa*. Front Microbiol 10:53. 10.3389/fmicb.2019.00053. 30787911 PMC6372532

[29] Pohl S, Klockgether J, Eckweiler D, Khaledi A, Schniederjans M, Chouvarine P, Tümmler B, Häussler S. 2014. The extensive set of accessory *Pseudomonas aeruginosa* genomic components. FEMS Microbiol Lett 356:235–241. 10.1111/1574-6968.12445. 24766399

[30] Dobrindt U, Hochhut B, Hentschel U, Hacker J. 2004. Genomic islands in pathogenic and environmental microorganisms. Nat Rev Microbiol 2:414–424. 10.1038/nrmicro884. 15100694

[31] Botelho J, Mourão J, Roberts AP, Peixe L. 2020. Comprehensive genome data analysis establishes a triple whammy of carbapenemases, ICEs and multiple clinically relevant bacteria. Microb Genom 6:mgen000424. 10.1099/mgen.0.000424. PMC7660259 32841111

[32] Agyepong N, Govinden U, Owusu-Ofori A, Essack SY. 2018. Multidrug-resistant gram-negative bacterial infections in a teaching hospital in Ghana. Antimicrob Resist Infect Control 7:37. 10.1186/s13756-018-0324-2. 29541448 PMC5845144

[33] Magiorakos A-P, Srinivasan A, Carey RB, Carmeli Y, Falagas ME, Giske CG, Harbarth S, Hindler JF, Kahlmeter G, Olsson-Liljequist B, Paterson DL, Rice LB, Stelling J, Struelens MJ, Vatopoulos A, Weber JT, Monnet DL. 2012. Multidrug-resistant, extensively drug-resistant and pandrug-resistant bacteria: an international expert proposal for interim standard definitions for acquired resistance. Clin Microbiol Infect 18:268–281. 10.1111/j.1469-0691.2011.03570.x. 21793988

[34] Poirel L, Naas T, Nordmann P. 2010. Diversity, epidemiology, and genetics of class D β-lactamases. Antimicrob Agents Chemother 54:24–38. 10.1128/AAC.01512-08. 19721065 PMC2798486

[35] Urbanowicz P, Izdebski R, Baraniak A, Żabicka D, Ziółkowski G, Hryniewicz W, Gniadkowski M. 2019. *Pseudomonas aeruginosa* with NDM-1, DIM-1 and PME-1 β-lactamases, and RmtD3 16S rRNA methylase, encoded by new genomic islands. J Antimicrob Chemother 74:3117–3119. 10.1093/jac/dkz262. 31211367

[36] Rådström P, Sköld O, Swedberg G, Flensburg J, Roy PH, Sundström L. 1994. Transposon Tn*5090* of plasmid R751, which carries an integron, is related to Tn*7*, Mu, and the retroelements. J Bacteriol 176:3257–3268. 10.1128/JB.176.11.3257-3268.1994. 8195081 PMC205496

[37] Shapiro JA, Sporn P. 1977. Tn*402*: a new transposable element determining trimethoprim resistance that inserts in bacteriophage lambda. J Bacteriol 129:1632–1635. 10.1128/JB.129.3.1632-1635.1977. 321437 PMC235145

[38] Hou Y-M. 1999. Transfer RNAs and pathogenicity islands. Trends Biochem Sci 24:295–298. 10.1016/S0968-0004(99)01428-0. 10431171

[39] Blattner FR, Plunkett G, 3rd, Bloch CA, Perna NT, Burland V, Riley M, Collado-Vides J, Glasner JD, Rode CK, Mayhew GF, Gregor J, Davis NW, Kirkpatrick HA, Goeden MA, Rose DJ, Mau B, Shao Y. 1997. The complete genome sequence of *Escherichia coli* K-12. Science 277:1453–1462. 10.1126/science.277.5331.1453. 9278503

[40] Wilderman PJ, Sowa NA, FitzGerald DJ, FitzGerald PC, Gottesman S, Ochsner UA, Vasil ML. 2004. Identification of tandem duplicate regulatory small RNAs in *Pseudomonas aeruginosa* involved in iron homeostasis. Proc Natl Acad Sci U S A 101:9792–9797. 10.1073/pnas.0403423101. 15210934 PMC470753

[41] Botelho J, Grosso F, Quinteira S, Brilhante M, Ramos H, Peixe L. 2018. Two decades of *bla*_VIM-2_-producing *Pseudomonas aeruginosa* dissemination: an interplay between mobile genetic elements and successful clones. J Antimicrob Chemother 73:873–882. 10.1093/jac/dkx517. 29373674

[42] Poirel L, Rodríguez-Martínez J-M, Al Naiemi N, Debets-Ossenkopp YJ, Nordmann P, 2010. Characterization of DIM-1, an integron-encoded metallo-β-lactamase from a *Pseudomonas stutzeri* clinical isolate in the Netherlands. Antimicrob Agents Chemother 54:2420–2424. 10.1128/AAC.01456-09. 20308383 PMC2876379

[43] Leski TA, Bangura U, Jimmy DH, Ansumana R, Lizewski SE, Li RW, Stenger DA, Taitt CR, Vora GJ. 2013. Identification of *bla*_OXA-51_-like, *bla*_OXA-58_, *bla*_DIM-1_, and *bla*_VIM_ carbapenemase genes in hospital *Enterobacteriaceae* isolates from Sierra Leone. J Clin Microbiol 51:2435–2438. 10.1128/JCM.00832-13. 23658259 PMC3697688

[44] Sun F, Zhou D, Wang Q, Feng J, Feng W. 2016. Genetic characterization of a novel *bla*_DIM-2_-carrying megaplasmid p12969-DIM from clinical *Pseudomonas putida*. J Antimicrob Chemother 10:909–912. 10.1093/jac/dkv426. 26679251

[45] Deshpande LM, Jones RN, Woosley LN, Castanheira M. 2014. Retrospective molecular analysis of DIM-1 metallo-β-lactamase discovered in *Pseudomonas stutzeri* from India in 2000. Antimicrob Agents Chemother 58:596–598. 10.1128/AAC.01541-13. 24145536 PMC3910751

[46] Farajzadeh Sheikh A, Shahin M, Shokoohizadeh L, Ghanbari F, Solgi H, Shahcheraghi F. 2020. Emerge of NDM-1-producing multidrug-resistant *Pseudomonas aeruginosa* and co-harboring of carbapenemase genes in South of Iran. Iran J Public Health 49:959–967. 32953684 PMC7475625

[47] Botelho J, Grosso F, Peixe L. 2017. Unravelling the genome of a *Pseudomonas aeruginosa* isolate belonging to the high-risk clone ST235 reveals an integrative conjugative element housing a *bla*_GES-6_ carbapenemase. J Antimicrob Chemother 3:77–83. 10.1093/jac/dkx337. 29029083

[48] Minakhina S, Kholodii G, Mindlin S, Yurieva O, Nikiforov V. 1999. Tn*5053* family transposons are *res* site hunters sensing plasmidal *res* sites occupied by cognate resolvases. Mol Microbiol 33:1059–1068. 10.1046/j.1365-2958.1999.01548.x. 10476039

[49] Kholodii GY, Mindlin SZ, Bass IA, Yurieva OV, Minakhina SV, Nikiforov VG. 1995. Four genes, two ends, and a *res* region are involved in transposition of Tn*5053*: a paradigm for a novel family of transposons carrying either a *mer* operon or an integron. Mol Microbiol 17:1189–1200. 10.1111/j.1365-2958.1995.mmi_17061189.x. 8594337

[50] Sajjad A, Holley MP, Labbate M, Stokes HW, Gillings MR. 2011. Preclinical class 1 integron with a complete Tn*402*-like transposition module. Appl Environ Microbiol 77:335–337. 10.1128/AEM.02142-10. 21037292 PMC3019745

[51] Lagatolla C, Edalucci E, Dolzani L, Riccio ML, De Luca F, Medessi E, Rossolini GM, Tonin EA. 2006. Molecular evolution of metallo-β-lactamase-producing *Pseudomonas aeruginosa* in a nosocomial setting of high-level endemicity. J Clin Microbiol 44:2348–2353. 10.1128/JCM.00258-06. 16825348 PMC1489503

[52] Marchiaro P, Viale AM, Ballerini V, Rossignol G, Vila AJ, Limansky A. 2010. First report of a Tn*402*-like class 1 integron carrying *bla*. J Infect Dev Ctries 4:412–416. 10.3855/jidc.1012. 20601796

[53] Roy Chowdhury P, Scott M, Worden P, Huntington P, Hudson B, Karagiannis T, Charles IG, Djordjevic SP. 2016. Genomic islands 1 and 2 play key roles in the evolution of extensively drug-resistant ST235 isolates of *Pseudomonas aeruginosa*. Open Biol 6:150175. 10.1098/rsob.150175. 26962050 PMC4821235

[54] de La Rosa JMO, Nordmann P, Poirel L. 2020. Pathogenicity genomic island-associated *crpP*-like fluoroquinolone-modifying enzymes among *Pseudomonas aeruginosa* clinical isolates in Europe. Antimicrob Agents Chemother 64:e00489-20. 10.1128/AAC.00489-20. 32340994 PMC7318011

[55] Abril D, Marquez-Ortiz RA, Castro-Cardozo B, Moncayo-Ortiz JI, Olarte Escobar NM, Corredor Rozo ZL, Reyes N, Tovar C, Sánchez HF, Castellanos J, Guaca-González YM, Llanos-Uribe CE, Vanegas Gómez N, Escobar-Pérez J. 2019. Genome plasticity favours double chromosomal Tn*4401b*-*bla*_KPC-2_ transposon insertion in the *Pseudomonas aeruginosa* ST235 clone. BMC Microbiol 19:1–12. 10.1186/s12866-019-1418-6. 30786858 PMC6381643

[56] Thrane SW, Taylor VL, Freschi L, Kukavica-Ibrulj I, Boyle B, Laroche J, Pirnay J-P, Lévesque RC, Lam JS, Jelsbak L. 2015. The widespread multidrug-resistant serotype O12 *Pseudomonas aeruginosa* clone emerged through concomitant horizontal transfer of serotype antigen and antibiotic resistance gene clusters. mBio 6:e01396-15. 10.1128/mBio.01396-15. 26396243 PMC4600120

[57] Battle SE, Rello J, Hauser AR. 2009. Genomic islands of *Pseudomonas aeruginosa*. FEMS Microbiol Lett 290:70–78. 10.1111/j.1574-6968.2008.01406.x. 19025565 PMC2648531

[58] Berger C, Rückert C, Blom J, Rabaey K, Kalinowski J, Rosenbaum MA. 2021. Estimation of pathogenic potential of an environmental *Pseudomonas aeruginosa* isolate using comparative genomics. Sci Rep 11:1370. 10.1038/s41598-020-80592-8. 33446769 PMC7809047

[59] Boyd EF, Almagro-Moreno S, Parent MA. 2009. Genomic islands are dynamic, ancient integrative elements in bacterial evolution. Trends Microbiol 17:47–53. 10.1016/j.tim.2008.11.003. 19162481

[60] Schmidt H, Hensel M. 2004. Pathogenicity islands in bacterial pathogenesis. Clin Microbiol Rev 17:14–56. 10.1128/CMR.17.1.14-56.2004. 14726454 PMC321463

[61] Hsiao WWL, Ung K, Aeschliman D, Bryan J, Finlay BB, Brinkman FSL. 2005. Evidence of a large novel gene pool associated with prokaryotic genomic islands. PLoS Genet 1:e62. 10.1371/journal.pgen.0010062. 16299586 PMC1285063

[62] Semsey S, Blaha B, Köles K, Orosz L, Papp PP. 2002. Site-specific integrative elements of rhizobiophage *16-3* can integrate into proline tRNA (CGG) genes in different bacterial genera. J Bacteriol 184:177–182. 10.1128/JB.184.1.177-182.2002. 11741858 PMC134759

[63] Larbig KD, Christmann A, Johann A, Klockgether J, Hartsch T, Merkl R, Wiehlmann L, Fritz H-J, Tümmler B. 2002. Gene islands integrated into tRNA^Gly^ genes confer genome diversity on a *Pseudomonas aeruginosa* clone. J Bacteriol 184:6665–6680. 10.1128/JB.184.23.6665-6680.2002. 12426355 PMC135438

[64] Hacker J, Blum-Oehler G, Muhldorfer I, Tschape H. 1997. Pathogenicity islands of virulent bacteria: structure, function and impact on microbial evolution. Mol Microbiol 23:1089–1097. 10.1046/j.1365-2958.1997.3101672.x. 9106201

[65] Buchrieser C, Brosch R, Bach S, Guiyoule A, Carniel E. 1998. The high-pathogenicity island of *Yersinia pseudotuberculosis* can be inserted into any of the three chromosomal *asn tRNA* genes. Mol Microbiol 30:965–978. 10.1046/j.1365-2958.1998.01124.x. 9988474

[66] Djapgne L, Panja S, Brewer LK, Gans JH, Kane MA, et al. 2018. The *Pseudomonas aeruginosa* PrrF1 and PrrF2 small regulatory RNAs promote 2-alkyl-4-quinolone production through redundant regulation of the *antR* mRNA. J Bacteriol 200:e00704-17. 10.1128/JB.00704-17. 29507088 PMC5915787

[67] European Committee on Antimicrobial Susceptibility Testing. 2017. Breakpoint tables for interpretation of MICs and zone diameters, version 7.1. http://www.eucast.org/fileadmin/src/media/PDFs/EUCAST_files/Breakpoint_tables/v_5.0_Breakpoint_Table_01.pdf.

[68] European Committee on Antimicrobial Susceptibility Testing. 2020. *Pseudomonas aeruginosa*: calibration of zone diameter breakpoints to MIC values, version 7.0. https://www.eucast.org/fileadmin/src/media/PDFs/EUCAST_files/Disk_criteria/Validation_2020/Pseudomonas_aeruginosa_v_7.0_January_2020.pdf.

[69] Bolger AM, Lohse M, Usadel B. 2014. Trimmomatic: a flexible trimmer for Illumina sequence data. Bioinformatics 30:2114–2120. 10.1093/bioinformatics/btu170. 24695404 PMC4103590

[70] Bankevich A, Nurk S, Antipov D, Gurevich AA, Dvorkin M, Kulikov AS, Lesin VM, Nikolenko SI, Pham S, Prjibelski AD, Pyshkin AV, Sirotkin AV, Vyahhi N, Tesler G, Alekseyev MA, Pevzner PA. 2012. SPAdes: a new genome assembly algorithm and its applications to single-cell sequencing. J Comput Biol 19:455–477. 10.1089/cmb.2012.0021. 22506599 PMC3342519

[71] Gurevich A, Saveliev V, Vyahhi N, Tesler G. 2013. QUAST: quality assessment tool for genome assemblies. Bioinformatics 29:1072–1075. 10.1093/bioinformatics/btt086. 23422339 PMC3624806

[72] Wick RR, Judd LM, Gorrie CL, Holt KE. 2017. Unicycler: resolving bacterial genome assemblies from short and long sequencing reads. PLoS Comput Biol 13:e1005595. 10.1371/journal.pcbi.1005595. 28594827 PMC5481147

[73] Seemann T. 2014. Prokka: rapid prokaryotic genome annotation. Bioinformatics 30:2068–2069. 10.1093/bioinformatics/btu153. 24642063

[74] Wood DE, Salzberg SL. 2014. Kraken: ultrafast metagenomic sequence classification using exact alignments. Genome Biol 15:R46. 10.1186/gb-2014-15-3-r46. 24580807 PMC4053813

[75] Alikhan NF, Petty NK, Ben Zakour NL, Beatson SA. 2011. BLAST Ring Image Generator (BRIG): simple prokaryote genome comparisons. BMC Genomics 12:402. 10.1186/1471-2164-12-402. 21824423 PMC3163573

