# Supplementary material for: Carbapenem Resistance Determinants Acquired through Novel Chromosomal Integrations in Extensively Drug-Resistant Pseudomonas aeruginosa
Source: Antimicrob Agents Chemother. 2021 Jun 17;65(7):e00289-21. doi: 10.1128/AAC.00289-21 (PMC8373256; doi:10.1128/AAC.00289-21)
Supplement: Supplemental file 1 — Supplemental Tables S1 to S3. Download AAC.00289-21-s0001.pdf, PDF file, 0.3 MB [file aac.00289-21-s0001.pdf]

Table S1 Patient data and source of specimens for the XDR *P. aeruginosa* isolates

**Strain ID**	**Collection time**	**Ward ID**	**Sex**	**Age group**	**Source**	**Diagnosis**
97	08/2015	A	F	10-20	Urine	Urinary tract infection
130	08/2015	B	F	20-30	Wound swab	Surgical site infection
140	09/2015	B	M	1-10	Wound swab	Surgical site infection
142	07/2015	C	F	1-10	Peritoneal fluid	Peritonitis

Table S2 *P. aeruginosa*[1] included in the phylogenetic and genome analyses

Strain ID	Assembly[2]	MLST[3]	*bla*DIM-1	*bla*IMP-1	Genomic Island (GI) tRNA-Pro(ggg)	Genomic Island (GI) sRNA PrrF1/PrrF2
KCRI-321A*[4]	GCF_900406895.1	244	+			
IOMTU 133*	GCF_001548335.1	1047	+			
NICED-PA-01*	GCF_009939155.1	1203	+			
1334/14	GCF_004193735.1	234	+			
97	GCF_002411865.3	234	+	+	PAGI-97A	PAGI-97B
130*	GCF_002411845.1	234	+	+	A-IIii[5]	GI[6]
140*	GCF_002411815.1	234	+	+	A-IIii	GI
142*	GCF_002411785.1	234	+	+	A-IIii	GI
P1_London_28_IMP_1_04_05*	GCF_001180345.1	111		+		
P2_London_28_IMP_1_06_05*	GCF_001180365.1	111		+		
P6_East_of_England_6_IMP_1_03_09*	GCF_001180525.1	111		+		
AR_0241*	GCF_003324405.1	111		+		
NCGM257*	GCF_001547955.1	357		+		
113*	GCF_004349425.1	664		+		
CCUG 59626*	GCF_008180925.1	664		+		
XDR-PA*	GCF_900707935.1	664		+		
AR_0103*	GCF_001874795.1	964		+		
VNMU144*	GCF_009887625.1	1047		+		
VNMU143*	GCF_009887715.1	1047		+		
NCGM1179*	GCF_000291745.1	1285		+		
12939*	GCF_002753655.1	1420		+		
NCGM2.S1*	GCF_000284555.1	na[7]		+		GI
CCUG 73744*	GCF_008244625.1	na		+		

[1] All circularized genomes with addition of DIM-1 or/and IMP-1-encoding genomes present in PATRIC (assessed April 16th, 2020)
[2] NCBI assembly accession number (https://www.ncbi.nlm.nih.gov/assembly)
[3] Multi Locus Sequence Type
[4] Genome not completed
[5] Phylogroup of *intG* carried by the GI integrated into the locus
[6] GI integrated into the sRNA PrrF1/PrrF2 locus of given
[7] Not assigned

W36662	GCF_001516185.2	17		
H26027	GCF_003798105.1	17		
early isolate NN2 (clone C)	GCF_900185255.1	17		
FA-HZ1	GCF_001750705.1	27	A-Iii	
HS9	GCF_003319235.1	27	A-Iii	
KRP1	GCF_009676885.1	27	A-Iii	
paerg000	GCF_900618245.1	27		
W45909	GCF_001516205.2	27		
AG1	GCF_009662315.1	111	A-IIi(c6)	GI
FRD1	GCF_000829885.1	111	A-IIi(c6)	
Carb01 63	GCF_000981825.1	111	A-IIi(c6)	
RIVM-EMC2982	GCF_002085605.1	111	A-IIi(c6)	
1	GCF_900497025.1	111	A-IIi(c6)	
F30658	GCF_001516265.1	111	A-IIii	
F5677	GCF_002946935.1	111		
AR445	GCF_003073735.1	111		
Y82	GCF_003369755.1	111		
RW109	GCF_900243355.1	111		
T63266	GCF_001516105.1	132		
IMP67	GCF_008033725.1	132		
IMP68	GCF_008033745.1	132		
IMP66	GCF_008033765.1	132		
Cu1510	GCF_001465155.1	137		
LESB58	GCF_000026645.1	146	A-Iii	
LES431	GCF_000508765.1	146	A-Iii	
12-4-4(59)	GCF_001482325.1	152		
S86968	GCF_001515845.2	155		
T38079	GCF_001515915.2	155		
F9670	GCF_001542835.1	155		
ATCC 27853	GCF_001618925.1	155		
Pa1207	GCF_002208645.1	155	A-IIi(c3)	
NCTC12903	GCF_900636755.1	155		
CF39S	GCF_011466835.1	175		
PcyII-29	GCF_902703185.1	175		
AR439	GCF_003073895.1	179		

FDAARGOS_532	GCF_003812165.1	179		
FDAARGOS_505	GCF_003813005.1	179		
RP73	GCF_000414035.1	198		
F63912	GCF_001594325.2	198		
YB01	GCF_009867055.1	231		
PA83	GCF_002215345.1	233	B	
AR_0230	GCF_002968695.1	233	B	
AR444	GCF_003073615.1	233	B	
AR_0110	GCF_003204335.1	233	B	
K34-7	GCF_003206535.1	233	B	
AR_0111	GCF_003571805.1	233	B	
W16407	GCF_001516165.2	244		
FDAARGOS_570	GCF_003813025.1	244		
Y89	GCF_003369735.1	245		
Y71	GCF_003408495.1	245		
E80	GCF_004291075.1	245		
FDAARGOS_610	GCF_006364795.1	252		
NCTC13359	GCF_901472545.1	252		
UCBPP-PA14	GCF_000014625.1	253		
M1608	GCF_001516365.2	253		
M37351	GCF_001516385.1	253		
L10	GCF_002223805.1	253		
H26023	GCF_003798145.1	253		
PA14Or_reads	GCF_900095805.1	253		
X78812	GCF_001542795.2	257		
RD1-3	GCF_009911735.1	257		
PAER4_119	GCF_001879525.1	260		
PA59	GCF_009497675.1	260		GI
paerg002	GCF_900618255.1	262		
paerg004	GCF_900618265.1	262		
paerg003	GCF_900618275.1	262		
paerg010	GCF_900618315.1	262		
paerg011	GCF_900618325.1	262		
paerg012	GCF_900618335.1	262		
AR_458	GCF_003288435.1	274		

A681	GCF_007559085.1	274		
PA1088	GCF_001792835.1	277		
PA8281	GCF_001792855.1	277		
PA11803	GCF_001792875.1	277		
PA7790	GCF_001870265.1	277		
Pa1242	GCF_002205375.1	277	A-IIi(c4)	
WCHPA075019	GCF_003052005.2	277		
PA298	GCF_005305005.1	277		
E90	GCF_008705235.1	282		GI-E90
JB2	GCF_003060845.1	296		
AR_455	GCF_003288355.1	298		
PABL048	GCF_003411785.2	298		
SCV20265	GCF_000510305.1	299		
B10W	GCF_001874465.1	308		
PASGNAM699	GCF_002104595.1	308		
PASGNAM345	GCF_002104615.1	308		
Pa58	GCF_002192495.1	308		
AR_0353	GCF_002968655.1	308		
Pa124	GCF_002192475.1	309	A-IIi	
Pa127	GCF_002205355.1	309	A-IIi	
paerg005	GCF_900618285.1	309		
paerg009	GCF_900618305.1	309		
PcyII-40	GCF_902703195.1	309		
BAMCPA07-48	GCF_001632245.1	313		
DN1	GCF_001722005.2	316		
Ocean-1155	GCF_002237405.1	316		
Ocean-1175	GCF_002237425.1	316		
C79	GCF_007833895.1	316		
PB369	GCF_002812825.1	319		
PB368	GCF_002812845.1	319		
B41226	GCF_003950235.1	357		
B17932	GCF_003952325.1	357		
BA7823	GCF_003626935.1	357	A-IIi (c5)	
BA15561	GCF_003713085.1	357	A-IIi (c7)	
SP4528	GCF_003716765.1	357	A-IIi (c7)	

SP4371	GCF_003950255.1	357	A-IIi (c7)	
B14130	GCF_003952305.1	357	A-IIi (c7)	
SP4527	GCF_003991465.1	357	A-IIi (c7)	
SP2230	GCF_003952285.1	357		
519119	GCF_007559105.1	360		
DK2	GCF_000271365.1	386		
SCVFeb	GCF_001900195.1	387		
SCVJan	GCF_001900225.1	387		
NHmuc	GCF_001900265.1	387		
DK1 substr. NH57388A	GCF_900069025.1	387		
H27930	GCF_001516325.2	389		
PA121617	GCF_001679685.1	389		
DHS01	GCF_000496455.2	395	A-IIi(c2)	
AR442	GCF_003073795.1	395	A-IIi(c2)	
CCUG 70744	GCF_003194245.1	395	A-IIi(c2)	
1811-13R031	GCF_009676765.1	395	A-IIi(c2)	
1811-18R001	GCF_009676785.1	395	A-IIi(c2)	
F22031	GCF_000816985.1	485		
PAO1	GCF_000006765.1	549		
ATCC 15692	GCF_001729505.1	549		
GIMC5015:PAKB6	GCF_003957825.1	549		
PAO1161	GCF_004102665.1	549		
FDAARGOS_767	GCF_006364735.1	549		
PAO1_Orsay	GCF_900070375.1	549		
PA_154197	GCF_002075065.1	550		
AR_0095	GCF_002997005.1	620		
IMP-13	GCF_003950015.1	621	A-Iii	
PAG5	GCF_011106815.1	639		
N15-01092	GCF_003571505.1	654	A-Ii	
PAK	GCF_902172305.1	693		
T2101	GCF_009720425.1	708		
60503	GCF_007559065.1	773		
ST773	GCF_009664165.1	773		
NCTC13715	GCF_900636975.1	773		GI
PA1	GCF_000496605.2	782		

PA1RG	GCF_001293085.1	782	
W60856	GCF_001516225.2	959	
PA99	GCF_009498355.1	964	A-Ii
MS14403	GCF_011045375.1	979	
AR_0356	GCF_002968755.1	1006	
AR441	GCF_003073695.1	1006	
B136-33	GCF_000359505.1	1024	
H25883	GCF_003798125.1	1076	
H47921	GCF_001516345.1	1105	
PA-3	GCF_003691345.1	1135	
LW	GCF_004010895.1	1182	
PA7	GCF_000017205.1	1195	
PcyII-10	GCF_900149285.1	1233	
PA_150577	GCF_002442855.1	1239	
PA34	GCF_003332705.2	1284	
F23197	GCF_001516245.2	1295	
C7-25	GCF_902703215.1	1710	
AR_0360	GCF_002968515.1	1712	
NCTC10728	GCF_900637045.1	1750	
NCTC9433	GCF_900478745.1	1920	
PA_D1	GCF_001721745.1	1971	
PA_D2	GCF_001721765.1	1971	
PA_D9	GCF_001721785.1	1971	
PA_D16	GCF_001721805.1	1971	
PA_D22	GCF_001721825.1	1971	
PA_D25	GCF_001721845.1	1971	
PA_D5	GCF_001722025.1	1971	
PA_D21	GCF_001722045.1	1971	
PABL017	GCF_003429205.1	2167	
AZPAE15042	GCF_006971785.1	2211	
N17-1	GCF_001606045.1	2362	
SJTD-1	GCF_000271985.2	2619	
8380	GCF_001548135.1	2619	
MTB-1	GCF_000504045.1	2689	
H5708	GCF_001516305.2	3050	

VA-134	GCF_001447845.1	3090		
NCTC13618	GCF_901472595.1	na	A-Iii	
M18	GCF_000226155.1	na		
PPF-1	GCF_002287725.2	na	A-IIi(c3)	
VRFPA04	GCF_000473745.2	na		GI
Y31	GCF_003369775.1	na	B	
PA1R	GCF_000496645.1	na		
YL84	GCF_000524595.1	na		
PAK	GCF_000568855.2	na		
NCGM 1984	GCF_000829255.1	na		
NCGM 1900	GCF_000829275.1	na		
DSM 50071	GCF_001045685.1	na		
F9676	GCF_001077475.1	na		
NCTC10332	GCF_001457615.1	na		
T52373	GCF_001516005.1	na		
USDA-ARS-USMARC-41639	GCF_001518975.1	na		
TRN6622	GCF_001921095.1	na		
E6130952	GCF_002085755.1	na		
Pa84	GCF_002205335.1	na		
PB353	GCF_002812865.1	na		
PB354	GCF_002812885.1	na		
PB350	GCF_002812905.2	na		
PB367	GCF_002812925.1	na		
AR_0354	GCF_002968585.1	na		
AR_0357	GCF_002968955.1	na		
CR1	GCF_003025345.2	na		
MRSN12280	GCF_003028335.1	na		
AR_0446	GCF_003193645.1	na		
AR_460	GCF_003288335.1	na		
PABL012	GCF_003429185.1	na		
24Pae112	GCF_003433235.1	na		
268	GCF_003641125.1	na		
FDAARGOS_571	GCF_003812885.1	na		
FDAARGOS_501	GCF_003812905.1	na		
AES1M	GCF_004355125.1	na		

AES1R	GCF_004355145.1	na
243931	GCF_007559125.1	na
CCUG 51971	GCF_008195485.1	na
HOU1	GCF_008245185.1	na
CFSAN084950	GCF_009648875.1	na
T2436	GCF_009720405.1	na
INP-43	GCF_009905195.1	na
VIT PC9	GCF_010694505.1	na
NCTC11445	GCF_900636735.1	na
NCTC13620	GCF_901472565.1	na

Table S3 Characteristics of integrons present in *P. aeruginosa* strain 97[1]

ID[2]	Chromosomal position	5'-CS[3]	Gene cassette arrays[2]	3'-CS
In1591	2630645- 2637503	*intI1Δ*	*qnrVC1, gcu165, aadA6*	*qacEΔ1, sul1, orf5/* IRt[4]
In1592	2637504- 2646449	*intI1*	*bla*$_{\text{DIM-1}}$, *gcu185*	*qacEΔ1, tni/* IRt
In1593	4853224- 4857408	IRi[5]/*intI1*	*dfrB5, aadA1*	*qacEΔ1, sul1, orf5Δ*
In1594	5155930- 5161775	*intI1*	*dfrB5, arr-2, dfrB5, dfrB5, bla*$_{\text{OXA-129}}$	*qacEΔ1, sul1, orf5/* IRt
In1595	5917750- 5926342	IRi/*intI1*	*bla*$_{\text{OXA-10}}$, *aacA4, bla*$_{\text{IMP-1}}$	*qacEΔ1, tni/* IRt

[1] GeneBank accession number: NZ_CP031449.2
[2] As given/identified by the Integral database (http://integrall.bio.ua.pt/)
[3] Conserved Sequence
[4] Inverted Repeat t, 25-bp conserved sequence: 5`-TGTCATTTTCAGAAGACGACTGCAC-3`
[5] Inverted Repeat I, 25-bp conserved sequence: 5`-GTGCAGCCGTCTTCTGAAAACGACA-3`
